# The influence of GMO media strategies on public perceptions of CRISPR crop technologies in Southern Ontario

**DOI:** 10.1080/21645698.2026.2620131

**Published:** 2026-02-11

**Authors:** Poornima Goudar, Alexander Hall

**Affiliations:** a Dalla Lana School of Public Health, University of Toronto, Toronto, Canada; b School of Interdisciplinary Life Science, McMaster University, Hamilton, Canada

**Keywords:** CRISPR crops, genetic engineering, genetically modified organisms (GMOs), public perceptions, public understanding of science, science journalism, science policy

## Abstract

Genetically modified organisms (GMOs) have often divided public opinion, one factor influencing perceptions of GMO technologies has been misunderstood or poorly communicated scientific messaging. However, advancements in gene editing tools like CRISPR/Cas9 offer new crop modification possibilities, prompting different regulatory frameworks than traditional GMO technologies. This research examines public understanding of GMOs, awareness of CRISPR crops, and how prior experiences with GMOs shape perceptions of new genetic technologies. A mixed-methods approach was employed, combining a public survey of adults in the Greater Toronto-Hamilton area and interviews with science journalists. Results show hesitance toward GMOs and CRISPR crops, with acceptance most impacted by consumer behavior and cost. Key interview themes include journalist’s concerns about levels of public education, the role of social media, and the cost of goods. Our findings suggest increased transparency and effective communication could improve public acceptance of GMOs and CRISPR crops. While CRISPR crops do not come under the GMO regulatory framework in Canada, our findings show that the public does not recognize this distinction. Without increased transparency and more effective communication CRISPR crops may become widely associated with the negative media frames that have shaped perceptions of GMOs.

## 1. Introduction

As food prices rise and climate change continues to affect agriculture, new strategies are being explored to address global food security. Over the past few decades, agricultural biotechnology and genetically modified organisms (GMOs) have been developed as ways to improve crop quality and yield.^1^ GMOs encompass microorganisms, plants, and animals, that have had their genetic material altered through transgenesis, a process where genes from other organisms are inserted, often using recombinant DNA (rDNA) techniques.^2–4^ Studies show that GMOs are safe, since their commercialization in 1994 they have generated controversy.^5,6^ As public debate grew, particularly regarding concerns about contamination and safety, the United Nation’s World Health Organization (WHO) and Food and Agriculture Organization (FAO) developed guidelines for GMO safety, prompting national regulations including those in Canada, overseen by the Canadian Food Inspection Agency (CFIA) and Health Canada.^7,8^

Within the past decade, various new genetic editing tools have been introduced. Of these, the clustered regularly interspaced short palindromic repeats (CRISPR-Cas9) system has emerged as a powerful tool for precise genetic editing in crops.^9,10^ These crops, commonly referred to as “CRISPR crops,” have the potential to enhance nutritional yield, improve pesticide resistance, and increase cost-effectiveness.^11,12^ CRISPR-based crop editing is often categorized under the broader label of “New Breeding Techniques” (NBTs), which is intended to encompass methods that modify crops without introducing foreign DNA, although no formal definition, criteria, or legal status currently exists for this classification.^4,13^

Both GMOs and CRISPR crops involve changing an organism’s DNA, however, the regulatory frameworks governing these technologies differ due to variations in their methodologies. While both processes can produce comparable outcomes, the key distinction lies in the introduction of external or foreign DNA.^2,3^ The CRISPR-Cas9 process acts like molecular “scissors” that break specific DNA sequences, triggering the cell’s natural repair mechanisms.^2^ This allows for the removal or addition of nucleotides without the incorporation of foreign DNA from another organism.^a^^a^It is important to note that CRISPR-Cas9 can still include the incorporation of foreign DNA if desired. These nucleotide modifications can lead to changes in the traits of organisms whether crops or animals, which has led some to consider CRISPR a more “natural” tool compared to GMOs.^2,3^ Furthermore, under Canada’s Guidelines for the Safety Assessment of Novel Foods, due to their well-characterized safety profiles, foods derived from plants that do not contain foreign DNA in the final product are exempt from the more extensive pre-market safety assessments designed for GMOs.^8^

While international regulations on genetically modified organisms (GMOs) vary widely,^14^ Canada does not require GMO labeling on consumer products.^8^ Although all novel foods, including GMOs, are subject to pre-market regulatory review, guidelines introduced in 2022 clarify that crops containing no foreign DNA in the final product are not subject to the same regulatory requirements as GMOs.^8,15^ As a result, gene-edited crops with no remaining foreign DNA are considered “indistinguishable” from those developed through conventional selective breeding or natural processes.^16^

Despite GMO regulation, there remains significant opposition to these technologies, which for some is connected to misunderstandings and negative media portrayals.^17^ In the late 1990s and early 2000s, GMOs were frequently framed in the media using terms such as “Frankenfoods” or “Franken-corn,” with some media outlets describing them as “dangerous” and comparing consumers to “laboratory animals.”^5,18,19^ While media discourse on GMOs has become less polarized and consumer concern has decreased slightly, significant resistance remains.^20^ Notably, in 2016 AquaBounty salmon became the first genetically modified animal approved for commercialization in the USA and Canada, marking a milestone in GMO regulation.^21^ Despite CFIA approval, which ensured all safety standards were met before AquaBounty salmon entered the market, critics including the Ecology Action Center, referred to this product as a “Frankenfish.”^22^ Here we see an example of media framing in action – the earlier pejorative terminology being used to quickly denote to readers that this Canadian environmental NGO is against this new product entering the market.^23^

Although Canadian regulatory bodies have established separate guidelines for GMOs and CRISPR crops, it remains unclear whether the public can effectively distinguish between these technologies. Given the historical opposition to GMOs, it is important to consider how past experiences may shape public attitudes toward CRISPR crops and impact their acceptance. In a national survey of 1049 Canadians, Charlebois et al.^21^ found that 52% of respondents were uncertain about consuming genetically engineered foods, with participants expressing greater comfort toward genetically modified (GM) fruits and vegetables than with animal-based products. Similarly, Williams et al.^24^ reported that Canadians have limited knowledge of modern plant breeding techniques and often express uncertainty about the benefits of GM crops. This lack of understanding is further illustrated by a 2016 Health Canada study, which found that although Canadians generally trust the national food safety system, most were unable to define the term “GMO.”^25^ This points to a broader issue of limited scientific literacy regarding genetic technologies.

More recently, attention has shifted toward public attitudes regarding new breeding techniques (NBTs), such as CRISPR-Cas9. Two large-scale online surveys^24,26^ examining perceptions of both GMOs and NBTs revealed a notable paradox: while many Canadians continue to express trust in food safety oversight, they remain skeptical of the benefits of the products resulting from these technologies. This tension between institutional trust and technological skepticism poses a significant challenge for the acceptance and integration of genetically engineered and edited products into Canada’s food system. Furthermore, it raises questions about the mechanisms that citizens have at their disposal to escalate valid concerns and the lack of forums for citizens to be active agents in any dialogue on the future of food-systems and health in Canada.

Internationally, several studies have examined consumer perceptions of CRISPR crops. A recent South Korean study examined public perceptions and acceptance of gene-editing technology and agricultural products.^27^ Using a national survey, Oh and Lee found uneven awareness of scientific terminology: familiar terms such as “gene scissors” were widely recognized, whereas CRISPR had low awareness.^27^ Willingness to purchase gene-edited products was higher than previously reported for GMOs, reaching 70%, and safety was identified as the most important factor in increasing social acceptance of genome-edited crops.^27^ Focusing on Costa Rica, Gatica-Arias et al.^28^ found that while understanding of CRISPR was low, over 80% of participants (*n* = 1,018) supported its use for nature conservation, disease treatment, and crop improvement. Similarly, Shew et al.^29^ conducted a multi-country study on willingness to pay (WTP) for hypothetical CRISPR rice versus GM rice, revealing that 51% of participants (*n* = 2,315) from the USA, Canada, Belgium, France, and Australia were open to consuming both. The study also highlighted that familiarity with GM technology positively influenced willingness to consume, with safety perceptions and environmental attitudes emerging as key factors, underscoring the complex relationship between public attitudes toward GMOs and CRISPR. Despite this previous literature, research regarding the public awareness and perception of CRISPR crops in Canada remains limited. While several studies have assessed attitudes toward GMOs and NBTs more generally, empirical data specifically addressing CRISPR crops and their comparative perception to GMOs is scarce.^21,25,30,31^ This knowledge gap is significant, especially given the differing regulatory frameworks for these technologies. Understanding consumer perceptions of CRISPR crops in relation to GMOs is essential for developing effective communication strategies. Additionally, the potential for misinformation as CRISPR becomes more integrated into food systems could lead to consumers conflating GMOs with CRISPR crops, (mis)shaping public communications, perceptions and ultimately acceptance.

Therefore, this study aims to explore how prior perceptions of GMOs may influence public perception of CRISPR crop technologies in Ontario, Canada. Specifically, the project sought to address the following research question: how have public communication and media strategies on GMOs influenced media coverage of CRISPR crop technologies, and in turn, shaped the public’s perception of them?

The objectives of this study were as follows:

1. Evaluate the current awareness and attitudes toward both GMOs and CRISPR crops among the population of Southern Ontario.
2. Determine whether public perceptions of GMOs correlate with perceptions of CRISPR crops.
3. Understand the factors influencing consumer WTP for CRISPR crops.
4. Gather first-hand stakeholder perspectives to anticipate potential trends in public acceptance of CRISPR crops, informed by past perceptions surrounding GMOs.

## 2. Materials and Methods

To address the study’s objectives a mixed-methods approach was deployed. A cross-sectional public survey was conducted to assess the current level of understanding, perceptions, and attitudes toward GMOs and CRISPR crops within the Greater Toronto-Hamilton Area (GTHA) in Southern Ontario, Canada. Subsequently, qualitative interviews were conducted with stakeholders, including science journalists, writers, and editors, to gain insights into their perspectives on these biotechnologies and to identify differences in the communication strategies they employ. This mixed-methods approach was designed to provide a comprehensive understanding of the research question and objectives. This project was approved by the McMaster Research Ethics Board (MREB) under project #7308.

## 3. Public Survey

### 3.1. Survey Development and Administration

The online survey instrument was developed based on a review of prior literature, with seven out of twenty-six measures being adopted from prior studies. The survey (Appendix A) ran from November 2024 to January 2025, was administered in English, and took approximately 10 minutes to complete. Convenience sampling was employed, with recruitment being conducted online using social media platforms including X (formerly Twitter), LinkedIn, and Instagram, as well as posters at McMaster University (Hamilton) and University Health Network sites at Toronto General Hospital, Toronto Western Hospital and Toronto Rehabilitation Institution. The survey was also shared through the research team’s networks using e-mail and direct messaging. Upon completion participants were encouraged to share the survey with other eligible participants, employing a form of virtual snowball sampling. We have mitigated potential bias in our convenience sample and improved transparency for readers by implementing the following verification methods: detailed methodology documentation, comparison with population benchmarks at both the Ontario and Canadian levels, multiple samples across various sites and locations, and the use of both quantitative and qualitative questions. A total of 406 individuals participated, and 333 complete responses were retained for analysis.

### 3.2. Survey Structure

The survey began with a landing page where participants provided their consent to take part in this study. In the opening section of the survey, we asked participants about their gender, age, GTHA region of residence, education, level of science education, and any active religious participation. Following these demographic questions, participants were asked to answer a series of measures about their food behaviors and perceptions of GMOs.

This section began with questions adopted from Charlebois et al.^21^ about participants’ diets and the top three factors they consider most important when grocery shopping. It then explored the participants’ familiarity with, attitudes toward, and media interactions regarding GMOs. Participants were provided with a lay summary of GMOs and asked about their feelings toward GMOs after reading it. An open-ended question was included to qualitatively capture the words participants associated with GMOs. The section finished with another measure from Charlebois et al.,^21^ which asked about participants’ willingness to consume GMO-based foods within specific produce categories.

In the final **s**ection of the survey, we switched to measuring perceptions of CRISPR-Cas9. Beginning with a lay summary of CRISPR crops, questions then assessed participants’ awareness, media interactions, and attitudes toward CRISPR crops. We included another open-ended word association question and then participants were asked about their understanding of the regulatory frameworks for both GMOs and CRISPR crops. Finally, using Likert scale questions adapted from Gatica-Arias et al.,^28^ participants’ willingness to consume CRISPR-edited foods was evaluated.

### 3.3. Data Analysis

Survey data was exported to Excel for manual cleaning and coding. Responses were assigned numerical values: single-answer questions were coded from 0 to 4, while multiple-select questions were binary-coded (0 = no, 1 = yes). The only exception was the demographic question about religion. Participants who selected multiple religions were consolidated into a new category, “2+ religions.” The cleaned data was statistically analyzed using R Studio, using the Chi-Square test and the Fisher Test functions, with a Monte Carlo Simulation using 10,000 replicates.

Word association responses were extracted, manually cleaned, and grouped into similar words (e.g., “Big” and “Bigger” were categorized as “Big”). The frequency of each word was recorded in an Excel spreadsheet and words that had a frequency of 1 were excluded from the final table. This table was then exported and used to generate a word cloud using an online tool at WordClouds.com.

## 4. Interviews with Science Communicators

Interviewees included journalists, editors, writers, and publishers with expertise in life sciences, agriculture, genetics, CRISPR, biotechnology, biomedical engineering, or synthetic biology. Both signed and verbal consent was gathered from interviewees. Semi-structured interviews were conducted via Zoom, with each lasting approximately 30 minutes and including six questions designed to explore the participant’s background, experiences, and perspectives on GMOs and CRISPR technologies (Appendix B). Data collection included an audio recording and transcription using Zoom’s automated tool. Interviewees could choose to decline the video interview and opt to respond via an online document instead. Transcripts were anonymized and emailed to interviewees for review, with participants having two weeks to redact any data they wished to exclude. The transcripts were manually cleaned, and a qualitative thematic analysis was carried out. Following Braun & Clarke,^32^ responses were consolidated, and similar themes identified throughout the interviews were grouped together.

## 5. Results

While for several key variables our sample was broadly representative of the GTHA’s demographics, on others there was significant divergence (Table 1). Notably, when compared to the 2021 Ontario census, our survey sample slightly overrepresented both women (65.17% vs 51% in Ontario Census) and individuals under 29 years of age.^33^ The sample is also skewed toward individuals with higher levels of education, with 73.27% of participants holding a bachelor’s or graduate degree (bachelor’s degree of higher: 36.8% in Ontario; 32.9% in Canada) and 69.97% of participants reporting their highest level of science education as a bachelor’s or graduate degree (Postsecondary certificate, diploma or degree in STEM: 22.1% in Ontario; 20.2% in Canada).^34^ Comparison with the census figures should only be taken as indicative, as the Statistics Canada data is for ages 25–64, while our participants were aged 18 + . The religious demographics of the participants align closely with those in the Ontario 2021 census, with Catholicism, other Christian denominations, Islam, and non-religious groups being the most common affiliations.^33^ It is important to note that since the survey was conducted only in English, the sample is limited to English-speaking individuals in the GTHA.

**Table 1.** Demographic results of survey participants.

	Number	Percentage (%)
Total	333	100
**Gender**		
Woman	217	65.17
Man	89	26.73
Non-Binary	14	4.20
Transgender Man	1	0.30
Transgender Women	1	0.30
Two-Spirit	1	0.30
Prefer Not to Say	5	1.50
No Response	5	1.50
**Age**		
18 years old − 29 years old	252	75.68
30 years old − 49 years old	54	16.22
50 years old − 69 years old	21	6.31
70 years or older	1	0.30
Prefer not to answer	2	0.60
No answer	3	0.90
**Level of Education**		
Some high school education	2	0.60
High school diploma	27	8.11
Some college but no diploma/degree	39	11.71
College degree/diploma	15	4.50
Bachelor’s degree	195	58.56
Graduate degree (Masters, PhD, MD, etc.)	49	14.71
Prefer not to answer	4	1.20
No answer	2	0.60
**Level of Science Education**		
High School	71	21.32
College	22	6.61
Bachelor’s Degree	198	59.46
Graduate Degree (Masters, PhD, MD etc.)	35	10.51
Prefer not to answer	4	1.20
No answer	3	0.90
**GTHA Region**		
Toronto Region	94	28.23
Halton Region	32	9.61
Peel Region	50	15.02
York Region	32	9.61
Durham Region	10	3.00
Hamilton Region	110	33.03
Prefer not to answer	4	1.20
No answer	1	0.30
**Religion**		
Protestantism	3	0.90
Catholicism	39	11.71
Christianity	34	10.21
Judaism	10	3.00
Islam	39	11.71
Buddhism	5	1.50
Hinduism	41	12.31
Inter/Non-Denominational	4	1.20
I do not actively participate in any religion	123	36.94
Prefer not to answer	8	2.40
Sikhism	3	0.90
Agnostic	2	0.60
Jainism	1	0.30
Orthodoxy	1	0.30
Taoism	1	0.30
Spiritual	1	0.30
Selected 2 or more	13	3.90
No answer	5	1.50
**Subjective Income**		
Many difficulties saving	42	12.61
Some difficulties saving	65	19.52
Able to save occasionally	109	32.73
Able to save regularly	79	23.72
Not sure/Prefer not to answer	37	11.11
	1	0.30

## 6. Descriptive Data

**Figure 1.**
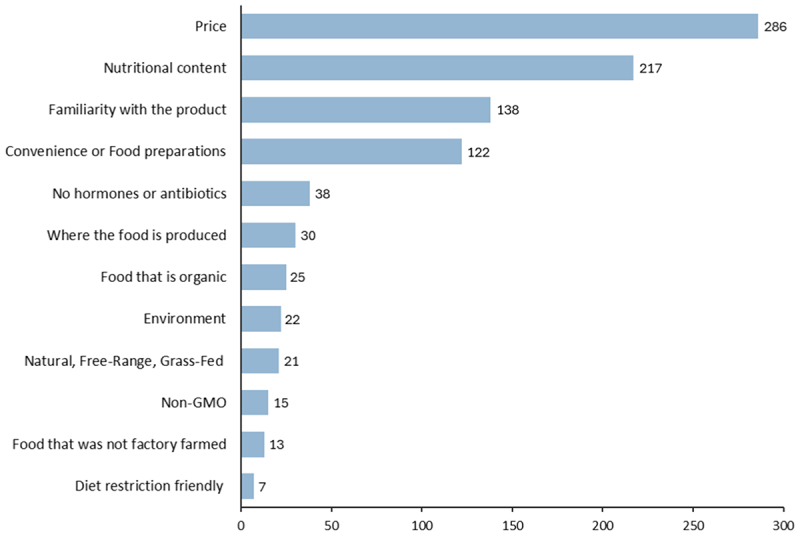
The top factors influencing food purchasing decisions.

Adapting from Charlebois et al.^21^ participants were provided with a list of factors influencing their food purchasing decisions and were asked to select their top three choices (Figure 1). The three most important factors identified were price (85.89%), nutritional content (65.17%), and familiarity with the product (41.44%). Notably, only 4.50% of participants ranked non-GMO as one of their top three most important factors when purchasing food.

Participants were also asked about their familiarity with, and attitudes toward, GMOs (Table 2). Most participants were familiar to some extent with GMOs, with the majority reporting they were “somewhat familiar” (28.61%). When asked about their feelings on the use of GMOs in agriculture, the majority of participants indicated they were neutral. Next, participants were provided with a lay summary about GMOs (Appendix C) and then asked again about their feelings toward GMOs in agriculture. After reading the lay summary, there was a decrease of 1.18% of responses in the “unacceptable” category, 2.34% in the “slightly unacceptable” category, and 8.33% in the “neutral” category. Conversely, there was an increase of 5.48% in the “moderately acceptable” category and 6.37% in the “perfectly acceptable” category.

**Table 2.** Assessing familiarity and attitudes of GMOs.

	Question	Not at all Familiar	Slightly Familiar	Somewhat Familiar	Moderately Familiar	Extremely Familiar
Familiarity	How familiar are you with Genetically Modified Organisms or GMOs?	32 9.64%	86 25.90%	95 28.61%	89 26.80%	30 9.04%
		Unacceptable	Slightly Unacceptable	Neutral	Moderately Acceptable	Perfectly Acceptable
Attitudes	How do you feel about the use of GMOs in agriculture?	32 9.61%	74 22.22%	108 32.43%	67 20.12%	52 15.62%
	After reading the summary, how do you feel about the use of GMOs in agriculture?	28 8.43%	66 19.88%	80 24.10%	85 25.60%	73 21.99%

**Figure 2.**
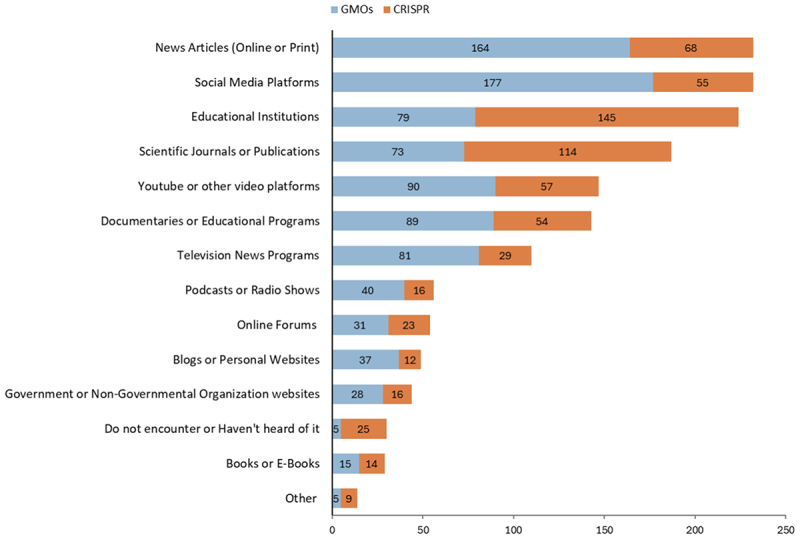
Types of media interaction with GMOs and CRISPR technologies.

Participants were asked about the nature of their interactions with GMOs and CRISPR-edited crops in the media. As shown in Table 3, the majority of participants engage with media content on GMOs on a rare (44.58%) or occasional (31.93%) basis. In contrast, interactions with CRISPR are more commonly reported as never (30.42%) or occasional (31.02%). Notably, there was a 17.77% increase in the number of participants who reported never interacting with CRISPR compared to GMOs. When asked about the types of media through which they engage with the topic, participants indicated that, in the case of GMOs, the primary sources of information are online or print news articles and social media platforms (Figure 2). In contrast, participants who have encountered information on CRISPR most commonly engage with it through educational institutions and scientific journals or publications. These trends perhaps reflect the relative newness of CRISPR technologies in relation to GMOs; however, we must also keep in mind the overrepresentation in our sample of respondents with a high level of science education, and those 29 and under.

**Table 3.** Frequency of media interaction with GMOs and CRISPR technology.

		GMOs	CRISPR	
	Count	Percent (%)	Count	Percent (%)
Never	42	12.65	101	30.42
Rarely (Few times a year)	148	44.58	61	18.37
Occasionally (Every few months)	106	31.93	103	31.02
Often (Monthly basis)	28	8.43	50	15.06
Always (Multiple times a week)	8	2.41	17	5.12

**Figure 3.**
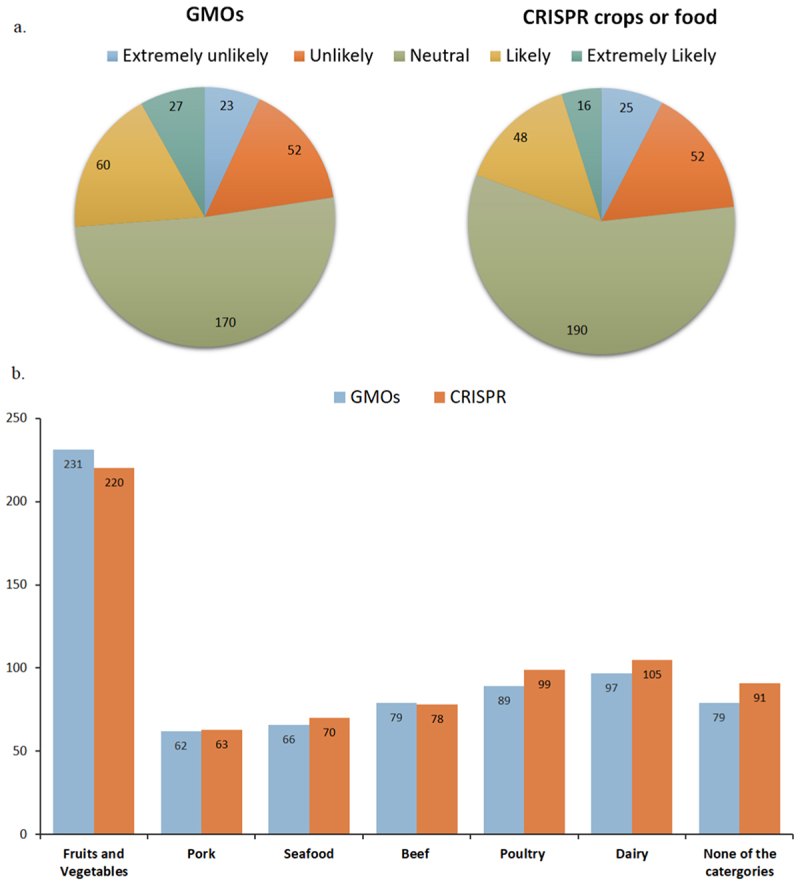
The likelihood of participants purchasing GMOs and CRISPR-edited crops or foods.

When participants were asked about their likelihood of purchasing GMOs (*n* = 332) and CRISPR-edited crops or food (*n* = 331), the distribution across the five Likert scale categories was similar for both technologies (Figure 3a). However, the “likely” and “extremely likely” groups were slightly larger for GMOs compared to CRISPR-edited crops. Participants who left this question unanswered were excluded from the analysis. Adapting a survey measure from Charlebois et al.,^21^ participants were then asked which food groups they would be willing to purchase edited foods from. Again, the likelihood of purchasing across these food categories were quite similar for both technologies (Figure 3b). Participants showed greater comfort with purchasing GMO and CRISPR-edited fruits and vegetables, with GMOs having a slightly higher number of responses. For animal-related food groups (including dairy), CRISPR-edited crops had a slight edge across all categories, except for beef, where GMOs had a negligible preference margin of one. Interestingly, CRISPR-edited crops had a higher number of participants selecting the “none of those categories” option compared to GMOs.

**Figure 4.**
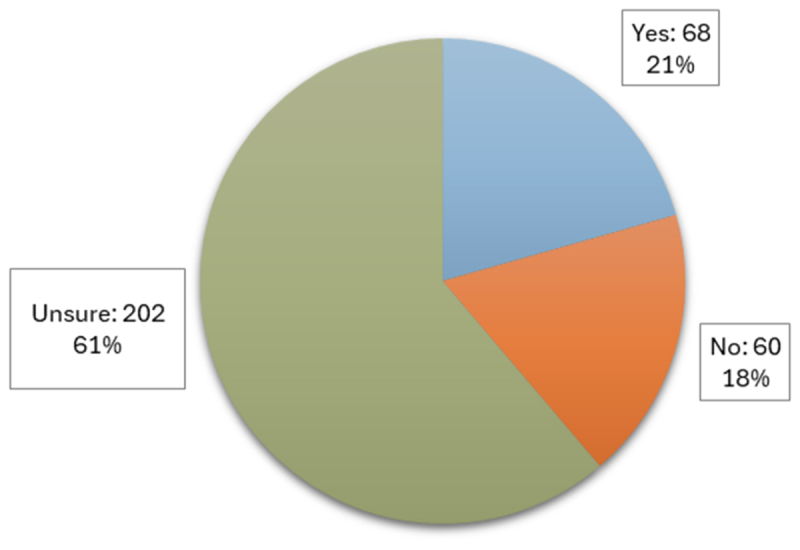
Participant views on whether GMOs and CRISPR-edited crops follow the same regulations in Canada.

To assess the public’s understanding of the regulatory frameworks governing these technologies, participants were asked whether they believed GMOs and CRISPR-edited crops were regulated in the same way (Figure 4, *n* = 330). The majority of participants (61%) were unsure if the two technologies followed the same regulations, while incorrectly 21% believed they did, and only 18% correctly identified that in Canada, GMOs and CRISPR crops do not fall under the same regulations.

**Figure 5.**
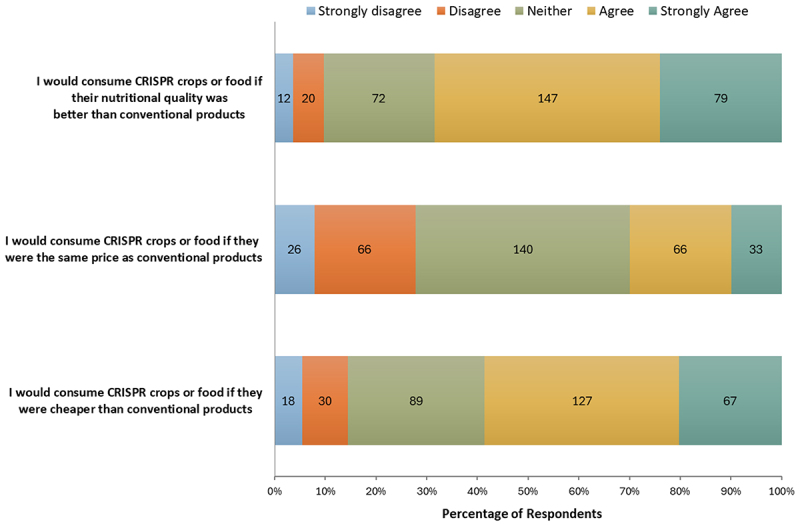
Participants’ willingness to consume CRISPR crops or foods.

Next, both consumer behavior and willingness to consume CRISPR-edited crops were assessed (Figure 5). Participants were given statements, adapted from Gatica-Arias et al.,^28^ describing various scenarios regarding participants’ willingness to consume CRISPR-edited crops or foods. When asked if participants would consume CRISPR crops if their nutritional quality was better than conventional products (*n* = 330), the response was predominantly positive, with 226 (68.48%) agreeing or strongly agreeing. However, this changed when participants were asked if they would consume CRISPR crops if they were priced the same as conventional products (*n* = 331). Now, 140 (42.30%) participants were neutral, with the remainder nearly equally split between disagree or strongly disagree (27.79%) and agree and strongly agree (29.91%). When participants were asked if they would purchase CRISPR-edited crops if they were cheaper than conventional crops (*n* = 331), there was a decrease in the negative and neutral responses and an increase in positive responses. When compared to if CRISPR-edited crops were the same price, the number of participants in the “strongly disagree” category decreased by 2.31%, “disagree” decreased by 10.88%, and “neutral” decreased by 15.41%. Conversely, the “agree” and “strongly agree” categories increased by 18.43% and 10.27%, respectively.

**Figure 6a.**
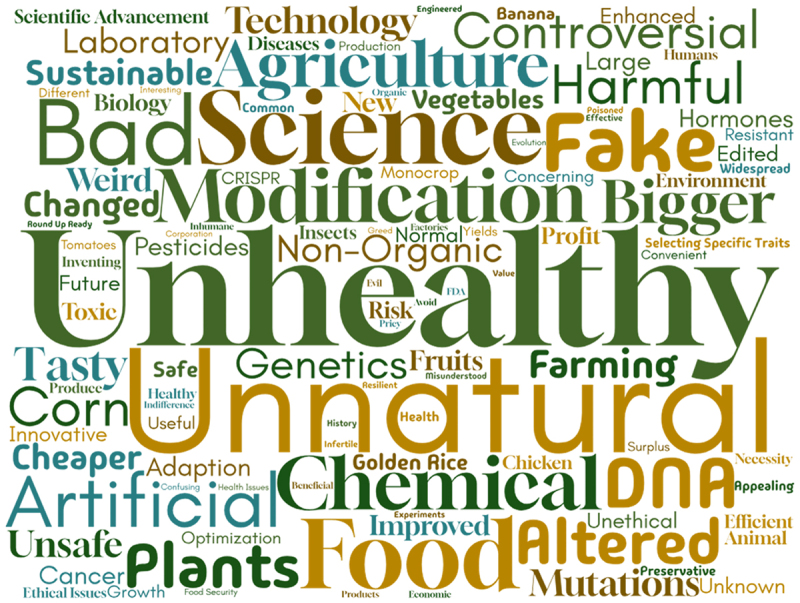
Word cloud for GMOs word association.

Finally, participants were asked to complete a word association task, providing the first three words that came to mind when they heard the terms “GMOs” or “CRISPR crops” (Figures 6a and 6b). The most frequently chosen words for GMOs were unhealthy, unnatural, bad, science, food, fake, artificial, chemical, and modification. For CRISPR crops, the most frequently chosen words were modification, GMO, gene editing, genes, DNA, science, new, unnatural, unknown, and genetics.**Figure 6b.**
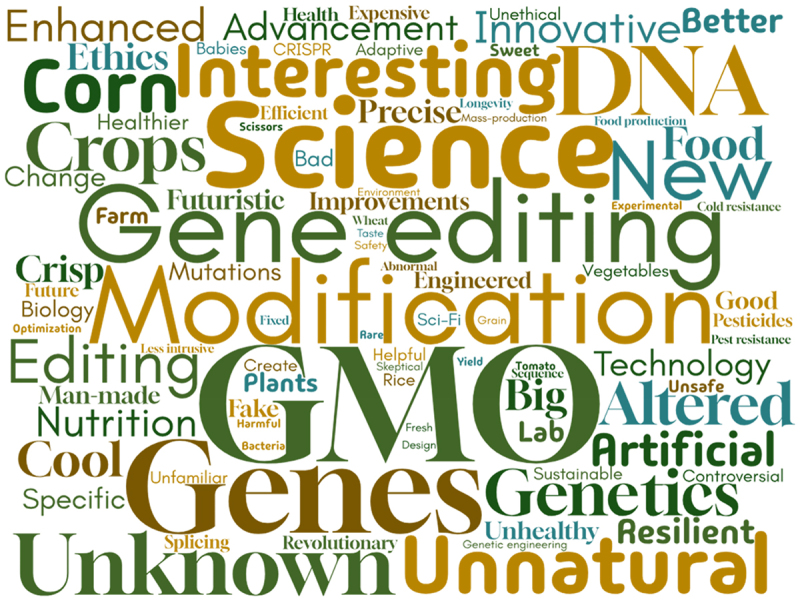
Word cloud for CRISPR crops word association.

## 7. Cross Tabulation Analysis

**Figure 7.**
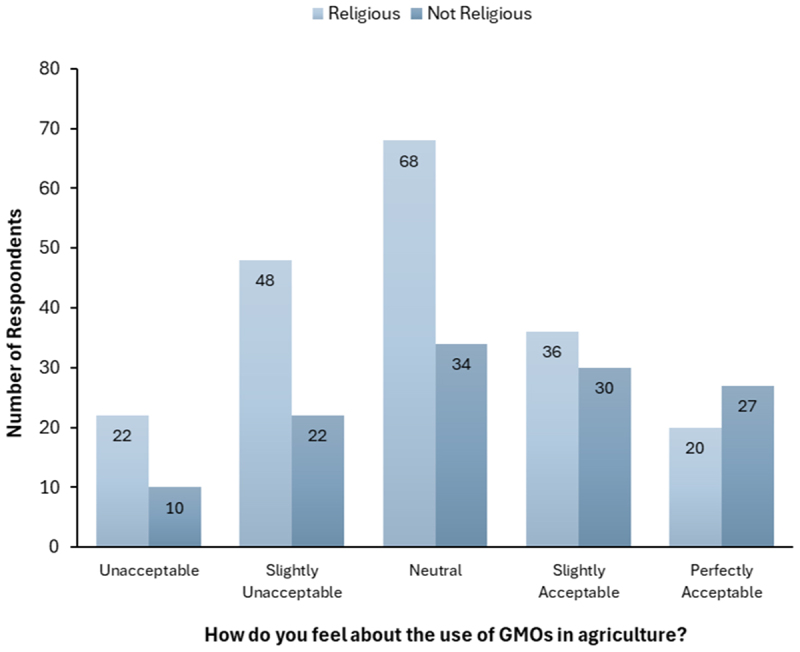
Participants’ opinions on the use of GMOs in agriculture in religious and non-religious participants.

To better understand any demographic factors that might potentially correlate with attitudes toward GMOs in agriculture, we conducted various cross-tabulations. When comparing religious affiliation and attitudes toward GMOs we found a statistically significant relationship (Figure 7). Religious participants (*n* = 194) were more likely to categorize the use of GMOs as unacceptable, slightly unacceptable, or neutral, while, non-religious participants (*n* = 123) had a more balanced distribution of responses, with a similar number of responses in the “slightly unacceptable” to “very acceptable” categories. Specifically, 36.08% of religious participants selected “unacceptable” or “slightly unacceptable,” compared to 26% of non-religious participants. Conversely, 28.87% of religious participants selected “acceptable” or “very acceptable,” compared to 46.34% of non-religious participants. Participants who preferred not to answer or who left the question blank were excluded from this analysis (*n* = 16). The chi-square test yielded a p-value of 0.02365 ( <0.05), indicating a statistically significant association between religion and attitudes toward GMOs.

**Figure 8.**
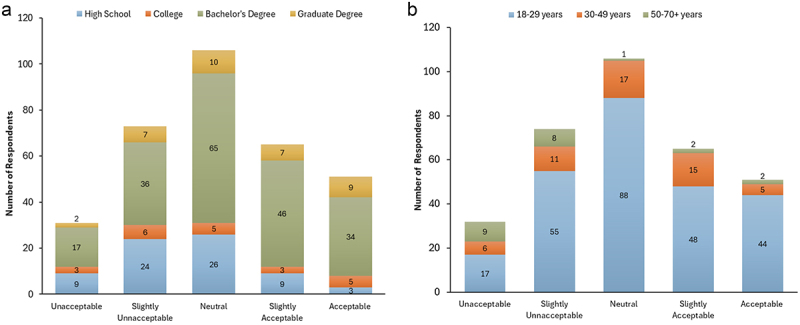
Attitudes toward the use of GMOs in agriculture among different levels of science education and age.

When comparing participants’ demographics – specifically their levels of science education and age – with their attitudes toward the use of GMOs (Figure 8), statistically significant relationships were observed. In Figure 8(a), the association between the levels of science education and attitudes toward the use of GMOs in agriculture were identified. The levels of science education included high school (*n* = 71), college (*n* = 22), bachelor’s degree (*n* = 198), and graduate degree (*n* = 35). Participants who selected “prefer not to answer” or left either question blank were excluded from the analysis (*n* = 3). A Monte Carlo simulation of Fisher’s Exact Test was performed, resulting in a p-value of 0.0211 ( < 0.05), indicating a statistically significant association between the levels of science education and attitudes toward GMOs. High school-educated individuals exhibited a more critical or uncertain stance, with 26 participants (36.62%) selecting “neutral,” and 24 participants (33.80%) selecting “slightly unacceptable.” Only 3 individuals (4.23%) from this group selected “acceptable,” suggesting a more hesitant or negative view within this category. In contrast, participants with a bachelor’s degree showed a more balanced distribution of responses, with 65 participants (32.83%) choosing the neutral category, followed by 46 (23.23%) choosing slightly acceptable, indicating a slightly more neutral/positive view on the issue. Participants with graduate degrees showed similar patterns, with 10 responses (25.57%) in the “neutral” and 9 (25.71%) responses in the “acceptable” categories. Two individuals (5.71%) selected “unacceptable,” suggesting a slightly more accepting or neutral view of the issue among this group.

In Figure 8(b), the association between the age of participants and attitudes toward GMOs was identified. The ages of the participants were 18–29 (*n* = 252), 30–49 (*n* = 54), and 50–70+ years (*n* = 22). Participants who selected “prefer not to answer” or left either question blank were excluded from the analysis (*n* = 5). A Monte Carlo simulation of Fisher’s Exact Test was performed, resulting in a p-value of 0.0002 ( < 0.05), indicating a statistically significant association between the participants’ ages and attitudes toward GMOs. Participants aged 18–29 display a varied response, with the majority (34.92%) selecting neutral, followed by 55 (21.83%) selecting slightly unacceptable and 48 (19.05%) selecting slightly acceptable. The 30–49 years age group displayed similar patterns, with the majority of respondents (31.48%) selecting neutral, followed by 15 (27.78%) selecting slightly acceptable, and 11 (20.37%) selecting slightly unacceptable. Different patterns were seen in participants aged 50–70+ years. 9 participants (40.91%) selected unacceptable, and 8 participants (36.36%) selected slightly unacceptable. Only 4 (18.18%) participants selected slightly acceptable or acceptable categories, suggesting a more negative view of GMOs within this age group.

## 8. Interview Data Results

Of the five interviews, with relevant science communicators and journalists (Table 4), four were online via Zoom and one via an online written submission.

**Table 4.** Interviewee background and experiences.

Interviewee	Profession and relevant work	Focus/Expertise	Background
**1**	Working on a research project focused on a specific biotechnology aimed at enhancing a biological process.	Biotechnology, GMOs, Gene Editing	Grew up in the agricultural community and pursued graduate training in science communication.
**2**	Freelance journalist covering food, agriculture, and health.	Science writing, Food, Agriculture, Health	Started in English then pursued Biology at the graduate level and then transitioned into science writing.
**3**	Documentaries and books on genetics, sports, and agriculture. Started an organization dedicated to raising awareness about genetics and biotechnologies.	Genetics, GMOs, Science Communication, Biotechnologies	Started with a humanities background, and later developed an interest in genetics, particularly within areas of sports and agriculture.
**4**	Works on creating educational resources and providing clear communication about GMOs and gene editing tools.	Science Writing, GMOs, Public Communication	Studied botany and shifted from lab work to science writing.
**5**	Freelance science journalist covering culture, nature, and the environment.	Science Journalism, Environment, Nature	Background in science writing, pursued science and environmental journalism.

Through qualitative thematic analysis of the interviews, several key themes emerged. The five most prominent are outlined below.

### 8.1. Elevating CRISPR in Comparison to GMOs

The media often portrays CRISPR as a superior alternative to GMOs, but interviewees emphasized how this narrative, risks perpetuating negative perceptions of GMOs while oversimplifying the complexities of both technologies. CRISPR is often depicted as a modern, exciting scientific breakthrough, while in reporting GMOs carry a stigma due to past negative media framing. Interviewee 2 explained the impact of these past media portrayals and their associated perceptions, “[i]f you just say ‘CRISPR’ to somebody … I think they would think, ‘Wow, it’s a super exciting recent scientific breakthrough,’ whereas I think if you say ‘GMO’ to somebody, they’ll be like, ‘Oh, I’m not supposed to buy those at the grocery store.’” Interviewee 1 further explained that this causes messaging to become segmented. When we say that certain kinds of biotechnology such as CRISPR crops are “better,” it is implied that there must be something wrong with other kinds of biotechnology, in this case, GMOs. Interviewee 3 further noted that this dichotomy between the two technologies is reinforced by scientists who strategically distance CRISPR from GMOs in an effort to avoid repeating the negative connotations and associations that became attached to GMOs. Interviewee 2 went on to explain that “scientists who know that it’s safe, throw GMOs under the bus because they don’t want to get fucked in the same way that GMOs were fucked.” This further highlights the risk of reinforcing the idea that GMOs are inherently problematic. While bringing GMOs down to elevate CRISPR may help the latter gain traction in the short term, it could also hinder efforts to foster a more nuanced understanding of these biotechnologies and their similarities and differences, ultimately affecting the public’s understanding of both in the long term.

### 8.2. Science Communication Industry

There has been a decline in specialized science journalism and in the digital age, the relationships between science communicators and their audiences have become more complex.^35^ Interviewee 1 noted that “media coverage of science is going to decrease because so many newsrooms if they exist anymore are laying off their science reporters or are no longer having a science beat or are competing with other types of science.” This statement highlights experts’ concerns about the growing challenge of maintaining specialized science journalism expertise, which is important for comprehension and nuance when covering technically complex subjects like biotechnology. As financial pressures mount and editorial priorities shift, many newsrooms are scaling back or eliminating specialized science coverage altogether. This trend potentially diminishes the public’s access to in-depth, and on occasion even accurate science reporting, making it more difficult for people to stay informed about scientific advancements. Interviewee 4 also reflected on this trend from a personal perspective, “[f]or [my entire department at a major college] in the US, I’m the only science writer.” This highlights the increased pressures on remaining science communicators, further straining the ability to produce comprehensive and well-informed science reporting. Furthermore, the decreasing number of science reporters often results in a single journalist covering multiple areas of science they may not be familiar with. As Interviewee 3 explained, “[m]any journalists covering environmental issues lack scientific backgrounds, particularly in fields like food science or nutrition, which leads to biased reporting.” They further explained that this can lead to skewed narratives, where sensational or controversial topics are prioritized over a more balanced presentation of the available scientific evidence. These systemic issues in science communication can impact reporting on nutrition, food, and biotechnology, ultimately affecting public knowledge and understanding.

### 8.3. Public Education and Transparency

Effective science communication requires addressing public misconceptions with empathy, transparency, and clear explanations. Interviewee 1 emphasized the importance of validating public concerns, even when they stem from misunderstandings. As they explained,

[Y]ou need to validate them [the public], even if it’s … “eating this genetically modified vegetable is going to mess with my DNA,” that is not something that happens, but that’s something that they believe. You need to … talk about how “No, this is how the technology works, and this is why we use the technology.”

This comment highlights the need for science communicators to acknowledge people’s fears and to guide them through the science using empathetic communication. Additionally, Interviewee 4 highlighted a lack of basic scientific understanding among the public, “every single year, students would be very surprised when they heard that all plants had DNA and that they were eating DNA every single day … that basic thing is a shock factor to people that scares them.” This underscores the limited understanding of fundamental concepts like DNA in plants, which can be startling for many. While, given that it may be difficult for all citizens to understand all of the complexities of biotechnologies, effective communication, grounded in transparency and patience, can be an essential component in improving knowledge and in turn fostering a more informed dialogue on GMOs. Interviewee 2, a science journalist with a background in food and science, shared how even those with relevant expertise can struggle to fully grasp the nuances between different genetic technologies. They explained, “[a] very quick Google search led me to believe that … GMOs have genetic material from an entirely different organism, while CRISPR crops would use genes from within that plant species … that nuance is something that was [previously] completely lost on me.” Given that CRISPR can also be used to insert genes from other species, this statement does not encompass the full range of CRISPR applications. Rather, the distinction they outlined actually reflects the divergent Canadian regulatory guidelines for GMOs and CRISPR crops, which draw distinction on the introduction of foreign DNA. Despite having a higher level of science literacy compared to the average citizen, Interviewee 2 and perhaps the search engine results they drew upon struggled to correctly navigate the distinctions in these genetic technologies and their applications. Again, this highlights the challenges that the broader public face in understanding these technologies. Together, these quotes highlight the critical role that our interviewees believed education and transparency must play in public biotechnology communications.

### 8.4. Utilizing Social Media

Social media has become a critical tool for disseminating scientific information, offering the potential to reach wide audiences quickly. Interviewees suggested that easy access to information, and the increase in social media influencers, makes it easier for misinformation to be spread, posing both challenges and opportunities. Some interviewees saw the potential for social media influencers to play a key role in shifting the narrative around biotechnologies. Interviewee 2 suggested that “you might need to have some social media influencers that are like, ‘Yeah, these CRISPR French fries are delicious.’” They argued that influencers could help make scientific concepts more relatable and engaging for the public, suggesting that this may be more effective than relying solely on professional journalists. On a similar note, Interviewee 4 emphasized the importance of not just combating misinformation but proactively sharing accurate and positive information. They stated, “[w]e’re reaching much fewer people, and we need to be a lot better about getting on social media, not just correcting because we don’t want to seem like we’re combating something but just putting good information out there.” Both interviewees highlighted a growing recognition that science communication needs to better adapt to the modern media landscape. Interviewee 1 shared similar sentiments, agreeing that social media is where science communicators need to “meet” the public, however, “it’s so easy to spread misinformation … [i]t’s so hard to prepare [researchers] for the blowback that comes and the trolls and the attacks.” They highlighted the difficulty of engaging with the public on platforms where misinformation can spread fast and where researchers often face negative feedback and outright hostility. Together, these insights highlight a dichotomy for science communicators on social media; while these platforms offer powerful opportunities to engage and inform the public, they also require strategic, thoughtful approaches to ensure that accurate information lands with the target audience.

### 8.5. Cost and Consumer Behavior

Interviewees all argued that economic factors, such as rising grocery prices and the premium cost of organic products, are likely to “influence” consumer acceptance of biotechnologies, including GMOs and CRISPR-edited foods. As affordability becomes a priority, consumers may prioritize cost over preferences for organic or non-GMO products, creating forced consumer interaction. Interviewee 3 critiqued the organic industry for appealing to consumers’ ideals and fears to justify higher prices, describing this as an “idealism margin.” They explained that consumers often buy organic products based on misconceptions, such as fearing GMOs cause health issues. However, as economic pressures grow, consumers may begin questioning whether the premium for organic products is truly worth the cost. Interviewee 1 further highlighted this possible shift, “as grocery prices go up, people aren’t going to have that choice, and they may want to eat organic or whatever, but they’re going to do what they can afford.” They argued that rising costs may shift consumer focus from the appeal of organic foods to what they can afford, even if that means turning to GMOs or CRISPR-edited foods. Together, these interviewees presented a seemingly logical argument about how economic pressures might reshape consumer behavior, even for those initially concerned with GMOs.

## 9. Discussion

Both GMOs and CRISPR crops involve alterations to an organism’s DNA, but the regulatory frameworks for these technologies differ in Canada due to variations in their processes.^8,15^ Despite this distinction meaning that the products created by these technologies receive different levels of regulatory scrutiny, it was unclear if the public could differentiate between them. Therefore, this study aimed to explore how past experiences with GMOs have influenced perceptions of CRISPR crop technologies within the population of the GTHA. With a specific focus on evaluating current awareness, attitudes, and consumer behavior toward both biotechnologies, this study also sought to gather first-hand stakeholder perspectives to predict potential trends that CRISPR crops may encounter informed by past patterns with GMOs. The results of our survey indicate that generally people in the GTHA have a more neutral or slightly negative attitude toward GMOs, while CRISPR technology is perceived more favorably, with greater emphasis on its novelty. Additionally, and perhaps crucially participants expressed greater interest in CRISPR-edited food when presented with additional accompanying benefits, such as a reduction in cost.

It was hypothesized that awareness and understanding of CRISPR crops would vary across the GTHA population, with higher levels of awareness expected among individuals with a background or training in science. Figure 8a shows a statistically significant (*p* = 0.0211 < 0.05) relationship between science education and attitudes toward GMOs. Individuals with university level science education (both bachelor’s and graduate degrees) were more likely to consider the use of GMOs in agriculture as “slightly acceptable” or “acceptable.” This may be related to their greater familiarity and understanding of the science involved, as exposure to the processes of biotechnologies could be linked to more positive views of GMOs. This further suggests that public education and transparency are important factors for how participants understand and perceive biotechnologies. Interview data supports this claim, with interviewees emphasizing the importance of transparency in their work engaging with the public. For example, Interviewee 1 noted that, in a class of non-science majors, most students were unaware that the food they consumed – like all plants – was made up of DNA. This highlights a basic knowledge gap that can hinder understanding of GMOs and CRISPR crops. While knowledge is not the only variable of importance within science-society relationships,^40^ it is unrealistic to expect the public to engage with the complexities of biotechnology without relevant foundational knowledge. This aligns with Figure 8a, which shows that individuals with less scientific education tend to have more negative or neutral attitudes toward GMOs, potentially due to lower familiarity with scientific concepts or interactions with biotechnologies.

Furthermore, when comparing age and attitudes toward the use of GMOs, more negative views were suggested by the answers in older respondents. While participants aged 18–29 and 30–49 displayed a more varied response, the majority of participants aged 50–70+ years selected unacceptable or slightly unacceptable as their categories. Genetic engineering is a relatively new field of science, and many older individuals may be less familiar with it, possibly contributing to increased negative attitudes.^41^ Additionally, the term “Frankenfoods” was first used in 1992, which means those aged 50–70+ may be more familiar with the term and the wider negative media framing it represents.^39^ In contrast, younger age groups may have had limited interaction with the term, the associated negative media framing, and the concerns represented in such coverage. These factors may help explain the more negative attitudes observed in the groups above.

Effective communication, grounded in transparency and patience, is important for addressing knowledge gaps in the general population in relation to biotechnologies. After participants were provided with a simple, neutral lay summary of GMOs, their attitudes toward GMOs in agriculture became more positive. The number of participants in the unacceptable, slightly unacceptable, and neutral categories decreased, while those in the acceptable and positive categories increased (Table 2). These results suggest that providing clear, accessible information about GMOs can improve public acceptance, reinforcing the relationship between familiarity with science and attitudes. While this survey used a short lay summary, it demonstrates that even a small amount of information can lead to a more positive reception of GMO technologies. These findings align with previous studies indicating that increased education about GMOs correlates with greater acceptance. Chrispeels et al.^42^ observed that non-science undergraduates had a more positive perception of GMOs after a peer-teaching session, and Hassan et al.^43^ reported an increase in GMO perception scores after participants attended a similar educational session. These results echo our findings, suggesting that providing clear, accessible information about GMOs correlates with increased public acceptance and reinforces the relationship between scientific education and attitudes. These same strategies could benefit gene-editing tools, including CRISPR crops.

However, a challenge arises from the growing disconnect between where the public receives information about science and its applications and where science communicators most commonly publish. When participants were asked about their media interactions with GMOs and CRISPR technology (Table 3), most encountered GMOs on a rare or occasional basis, while most interacted with CRISPR on an occasional basis or never. However, the key difference observed lies in the nature and location of these interactions. Participants primarily encountered GMOs through social media and news articles, while CRISPR was mainly encountered through scientific publications and academic institutions (Figure 2). While this could be an artifact of the overrepresentation of those with a science degree in our sample, nonetheless it highlights the challenge for science communicators in bringing new biotechnologies into media platforms and formats where the majority engage with such technological applications. As gene editing advances, science communicators must adapt their strategies to address shifting media consumption patterns. Many interviewees noted the need for the industry to “do a better job of getting onto social media” and that “reaching the public now requires more than well-crafted articles in well-regarded publications.” Interviewees emphasized that they are reaching fewer people and that, in addition to combating misinformation, they also need to share new information in engaging ways through social media. While these increasing responsibilities come at a time when science journalists are facing significant challenges, such as increased career uncertainty, the rapidly shifting media landscape also presents opportunities. For example, studies have shown that collaboration among science journalists and news organizations is increasing and that the social media ecosystem allows scientists to bypass gatekeepers such as journalists and legacy media corporations.^8,15,16,35^

Moreover, we know from prior studies that attitudes toward GMOs and their use in agriculture not only vary across levels of science education but also are significantly shaped by other demographic and identity markers. When comparing religious and non-religious participants (Figure 7), we found that religious participants were more likely to find GMOs “unacceptable” or “slightly unacceptable” compared to their non-religious counterparts. These findings align with previous studies, as religious concerns have long been a significant barrier to the adoption of GMOs, particularly in developing countries.^44^ Research by Heiman et al.^45,46^ found that in general religious individuals were more opposed to GM foods, while secular individuals tended to be more supportive. Adding more nuance to our understanding of how religious belief intersects with personal acceptance of GMO technologies, Ghasemi et al.^38^ found that people with stronger religious beliefs were more likely to view GM foods negatively. Several reasons may explain these results, including the belief among some religious participants that GMO crop production conflicts with their religious views, such as interfering with God’s creation, or being morally unacceptable.^38^

Furthermore, we expected that consumers were more likely to purchase CRISPR-edited food if it offers improved nutritional benefits and/or a lower price point. We saw that most participants had a neutral stance on purchasing GMOs and CRISPR-edited crops (Figure 3), with 51.20% selecting neutral for GMOs and 57.40% for CRISPR crops. This suggests that, without specific advantages, consumers do not have a strong preference for either option. Figure 5 further supports this hypothesis, showing that 44.55% of participants would consume CRISPR crops if they offered better nutritional quality, and 38.37% would purchase them if they were cheaper than conventional products. Notably, the neutral response rate decreased significantly in these questions compared to Figure 3. Only 21.82% of participants responded neutrally about consuming CRISPR crops if there was better nutrition, and 26.89% responded neutrally about purchasing CRISPR crops if there was a lower cost. This indicates that when specific benefits, such as improved nutrition or lower prices are offered, consumers find CRISPR-edited crops more appealing. These findings align with a study by Gatica-Arias et al.,^28^ which found that consumers in Costa Rica were more inclined to purchase CRISPR-edited rice or beans if they were priced lower than conventional options. Additionally, previous studies have shown that consumers are more likely to buy GM and gene-edited products when offered at discounted prices.^29,31,47^

This insight is crucial for science communicators and other stakeholders alike, emphasizing the need to effectively convey tangible benefits like improved nutrition and lower prices. By clearly communicating these advantages, alongside improved educational content science communicators can help inform public debate, and in turn help consumers make better informed decision with regards to gene-edited food products. The importance of this finding is further reinforced by the fact that participants indicated that cost, nutritional quality, and product familiarity were the most important factors when purchasing groceries (Figure 1). Infact, only 4.5% of participants ranked “non-GMO” as an important factor when purchasing food. Similar results have been observed in previous studies: Charlebois et al.^21^ found that nutritional content and familiarity were among the top four factors, while Lusk and Briggeman^48^ identified safety, nutrition, taste, and price as the top food values. More recently, both Vasquez et al.^26^ and Macall et al.^49^ reported similar findings in Canada, where nutrition, price, place of origin, and taste were deemed the most important food values. These results highlight that price and nutritional content consistently emerge as key factors in consumer decision-making.

It is important to note that the most common response related to income in our sample was “able to save occasionally” (32.73%), suggesting that cost-saving options are particularly appealing to many participants. Economic factors, such as rising grocery prices and the premium cost of organic products, are likely to impact consumer acceptance of biotechnologies. Interviewees also reinforced this, emphasizing that given these rising costs, consumers will likely prioritize affordability, even if it means choosing GMOs or CRISPR-edited foods, as seen in Figure 5. These economic pressures continually reshape consumer behavior, which may create an opportunity to highlight gene editing and CRISPR technology as cost-effective and nutritionally beneficial options.

Despite their differing regulatory regimes, participants associated similar words with both GMOs and CRISPR crops (Figures 6). Common terms such as “unnatural,” “science,” and “modification” were frequently associated with both technologies, suggesting that the public sees some level of similarity. Interestingly, “GMOs” also appeared as a commonly associated word for CRISPR crops, highlighting that popular definitions of what constitutes a genetically modified organism diverge from their distinct interpretation under the Canadian regulatory framework. However, it is important to note that the GMO questions and lay summary were presented prior to the CRISPR crop word association, which could have introduced a priming effect. When analyzing the word associations for GMOs, negative connotations dominate, with words like “unhealthy,” “unnatural,” “bad,” “fake,” and “artificial” frequently cited. These negative associations suggest that public perceptions of GMOs are still largely characterized by skepticism, fear, or distrust, reflecting broader concerns about safety, ethics, and environmental impacts. In contrast, word associations for CRISPR crops were more neutral or positive, with terms like “gene editing,” “advancement,” “science,” and “new” appearing more frequently. Although “unknown” and “unnatural” were also still present, these words had a lower frequency. This suggests that CRISPR crops are viewed more neutrally, albeit with some associated uncertainties. Although framed more neutrally, the overlap in word associations between CRISPR crops and GMOs suggests that negative perceptions of GMOs could influence views on CRISPR crops. As participants primarily encounter information about GMOs through news articles and social media, which presents information in varying ways (Figure 2), media framing is a crucial component that shapes these perceptions. Social media is largely unmoderated, allowing for a wide range of content, which can vary in quality and veracity, while news articles often feature personal opinions and sometimes adopt sensationalized or contentious tones when discussing these technologies. In contrast, in our study CRISPR was more commonly encountered through scientific publications and academic institutions where oftentimes, neutral language is the expectation.^36,50^ These differences between CRISPR and GMOs that we observed in media interaction, are also evident in the word clouds generated for both technologies (Figure 6).

Although the word association results indicate some overlap in how consumers perceive GMOs and CRISPR crops, our wider data shows that perceptions of the two technologies do diverge significantly. This distinction is also evident in the actions of some scientists, who, despite knowing GMOs are scientifically safe, distance themselves from the term to avoid its negative associations. One interviewee explained that scientists who are aware of GMOs’ safety, downplay them at conferences to elevate CRISPR crops, fearing a backlash similar to that which GMOs faced. However, by emphasizing the benefits of CRISPR while downplaying GMOs, there is a risk of reinforcing the perception that GMOs are inherently problematic despite evidence supporting their safety. This is of even more concern, given that we have identified that despite the regulatory divergence, for many citizens there is not a clear difference between the two technologies, with a significant number of respondents assuming that CRISPR crops are a form of GMO. Wherever these definitional boundaries fall, GMOs and CRISPR technologies are both used in agriculture and emphasizing the superiority of one over the other may shape perceptions and discussions in misleading ways.^51,52^

## 10. Conclusion

This study aimed to explore how prior perceptions of GMOs may influence public perception of CRISPR crop technologies within Southern Ontario. Specifically, this study sought to understand the current awareness, understanding, and perception of these biotechnologies, the factors influencing WTP for CRISPR crops in the region, and based on stakeholder perspectives the potential challenges CRISPR crops may face in their adoption by consumers. The findings reveal that the populace still holds a negative perception of GMOs, while CRISPR crops were more associated with a neutral or slightly positive stance. Additionally, our data suggests that the public would need to see added nutritional benefits and/or lower costs to consider buying CRISPR crops over conventional crops. Lastly, stakeholders identified both challenges and opportunities for future media trends regarding CRISPR crops. These included systemic issues within the science communication industry, the difficulties and importance of engaging the public in an empathetic and transparent manner, the dichotomy of social media as both removing barriers and increasing challenges, the impact of consumer behavior on food choices, and the potential risks associated with positioning CRISPR crops as superior to GMOs.

The results of this study should be interpreted with the following limitations in mind. The survey used virtual snowball sampling, which may have affected the representativeness of the sample and introduced potential bias.^53^ Additionally, the sample contains a disproportionate number of participants in the 18–29 age group and a slight skew toward individuals with higher levels of education.^33^ Further, the reliance on single-item measures in the survey limits the scope of measurement and may reduce the ability to fully capture the breadth and complexity of perceptions and factors influencing attitudes toward GMOs and gene-edited crops. It is important to note that variables such as political ideology and trust in science, which are known to influence perceptions of emerging technologies, were not measured due to the limited scope and timeframe of the study. Finally, despite only interviewing 5 stakeholders, the range and expertise of the professionals interviewed, means we are confident about the generalizability of the themes identified by this qualitative component.

In developing this research further, we would improve and refine the survey measures to increase their effectiveness. For example, in prioritizing brevity and accessibility in the survey design, we defined GMOs as modified varieties whose DNA has been changed, but this is true for every new crop variety that is approved and grown for food consumption in Canada. It is the mechanism of how this DNA was changed that delineates GMO from other crop-development processes, such as selective breeding. The lack of clear transparent definition here could have potentially biased responses toward increased concern about GM and gene-edited varieties.

Future research in Ontario and beyond, should target additional surveys at specific demographic groups, such as religious individuals or those with limited science education, to gain a deeper understanding of their perceptions of biotechnology. Future studies should also focus on older Ontarians, who were underrepresented in this study. Moreover, focus groups could provide valuable insights into the factors influencing attitudes and perceptions, particularly regarding the negative associations identified and the social dynamics and pressures that effect purchasing decisions. This approach would help uncover the underlying reasons behind these associations and offer context on participants’ motivations and thought processes, yielding important insights about more effective communication strategies regarding biotechnologies. Finally, it is recommended that future studies actively engage with relevant social psychological literature, such as heuristics and related concepts including the “cognitive miser” and “motivated tactician.”^37^ Although implicitly informing the research design in this study, more explicit engagement with these frameworks, as they apply to science communication would help further develop, focus, and refine measures exploring the influence of aspects including ideological predispositions, the influence of mass media, and stereotyping.

## Supplementary Material

Supplemental Material
