# Supplementary material for: The influence of GMO media strategies on public perceptions of CRISPR crop technologies in Southern Ontario
Source: GM Crops Food. 2026 Feb 11;17(1):2620131. doi: 10.1080/21645698.2026.2620131 (PMC12915814; doi:10.1080/21645698.2026.2620131)
Supplement: Supplemental Material [file KGMC_A_2620131_SM6060.docx]

**Appendix A**

**Survey Instrument:**

**Section 1: Demographic Information**

### How do you self-identify in terms of gender?

- Man
- Woman
- Non-binary
- Prefer to self-identify
- Transgender Man
- Transgender Woman
- Two-spirit
- Prefer not to say
- Other

What is your age?

- 18 years old - 29 years old
- 30 years old - 49 years old
- 50 years old - 69 years old
- 70 years or older
- Prefer not to answer

### What is your highest level of education? (currently enrolled or completed)

- Some high school education
- High school diploma
- Some college but no diploma/degree
- Associate's degree
- College degree/diploma
- Bachelor's degree
- Graduate degree (Masters, PhD, MD, etc.)
- Prefer not to answer

### What is your highest level of science education? (currently enrolled or completed)

- High School
- College
- Bachelor's Degree
- Graduate Degree (Masters, PhD, MD etc.)
- Prefer not to answer

Where do you reside in the GTHA?

- Toronto Region
- Halton Region
- Peel Region
- York Region
- Durham Region
- Hamilton Region
- Prefer not to answer

### Do you actively participate in any of the following religions? (Select all that apply)

- Protestantism
- Catholicism
- Christianity
- Judaism
- Islam
- Buddhism
- Hinduism
- Inter/Non-denominational
- I do not actively participate in any religion
- Prefer not to answer
- Other:

### What is your individual subjective income? Do you experience:

- Many difficulties saving
- Some difficulties saving
- Able to save occasionally
- Able to save regularly
- Not sure/Prefer not to answer

**Section 2: Food Behaviours and Perceptions of GMOs**

### From the following list, please select 3 options that are the most important to you when purchasing food:

- Price
- No hormones or antibiotics
- Nutritional content
- Familiarity with the product
- Where the food is produced
- Non-GMO
- Environment
- Natural, Free- Range, Grass-Fed
- Convenience / Food Preparations
- Food that is organic
- Food that was not factory farmed
- Other:

### Which of the following best describes your current diet? (Select all that apply)

- Vegetarian
- Pescatarian
- Vegan
- Omnivore
- Gluten-Free
- Carnivore
- Keto
- Paleo
- Mediterranean
- Prefer not to answer
- Other:

### How familiar are you with Genetically Modified Organisms or GMOs?

- Not at all familiar
- Slightly familiar
- Somewhat familiar
- Moderately familiar
- Extremely familiar

### How do you feel about the use of GMOs in agriculture?

- Unacceptable
- Slightly unacceptable
- Neutral
- Slightly acceptable
- Perfectly acceptable

Here is a brief description of GMOs provided for your reference:

Genetically Modified Organisms (GMOs) are plants or animals whose DNA has been changed using various techniques to improve certain traits. These methods often involve introducing genes from different species into the organism to enhance qualities like pest resistance or nutritional value. This allows scientists to create crops that can grow better and adapt to environmental challenges.

After reading that summary, how do you feel about the use of GMOs in agriculture?

- Unacceptable
- Slightly unacceptable
- Neutral
- Slightly acceptable
- Perfectly acceptable

### How often do you encounter and/or interact with media regarding GMOs?

- Never
- Rarely (Few times a year)
- Occasionally (Every few months)
- Often (Monthly basis)
- Always (Multiple times a week)

### Where do you primarily receive information or encounter GMOs in the media? Please select all that apply

- News articles - online or print (e.g., CBC, National Post, Toronto Star etc.)
- Television news programs
- Documentaries or educational programs
- Social media platforms (e.g., Facebook, X (Twitter), Instagram, Reddit, Linkedin etc.))
- Blogs or personal websites
- Podcasts or radio shows
- Scientific journals or publications
- Online forums or discussion groups
- Government or Non-Governmental Organization (NGO) websites
- Educational institutions (e.g., university websites, lectures)
- YouTube or other video platforms
- Books or e-books
- Other:

### To what extent do you believe media coverage has shaped your views on GMOs?

- Not at all
- A little
- Somewhat
- Quite a bit
- Very much

When you hear the words “GMOs” or “Genetically Modified Organisms,” please write down the first three words that come to mind. If you are unable to think of three, please provide as many as you can.

Where available, how likely are you to purchase genetically modified foods when shopping?

- Extremely unlikely
- Unlikely
- Neutral - Neither unlikely nor likely
- Likely
- Extremely likely

### Would you purchase genetically modified foods from the following categories? Please select all that apply:

- Seafood
- Fruits and Vegetables
- Dairy
- Poultry
- Beef
- Pork
- None of the above or I would not purchase

**Section 3: Perceptions of CRISR**

Here is a brief description of CRISPR crops provided for your reference:

CRISPR/Cas9 is a gene-editing tool that allows scientists to make precise changes to DNA. It targets specific parts of the genetic code and cuts or modifies the DNA at those spots. This technology can improve crops by enhancing traits like nutritional value or pest resistance, offering a new way to address challenges in food production.

### How much have you heard or read about gene editing via CRISPR/Cas-9?

- Never
- Rarely (heard/read about it a few times)
- Occasionally (heard/read about it multiple time through the year)
- Often (heard/read about it on a monthly basis)
- Always (heard/read about it multiple times a week)

### Where have you encountered/learned about gene editing via CRISPR /Cas-9? Please select all that apply:

- News articles - online or print (e.g., CBC, National Post, Toronto Star etc.)
- Television news programs
- Documentaries or educational programs
- Social media platforms (e.g., Facebook, X (Twitter), Instagram, Reddit, Linkedin etc.))
- Blogs or personal websites
- Podcasts or radio shows
- Scientific journals or publications
- Online forums or discussion groups
- Government or Non-Governmental Organization (NGO) websites
- Educational institutions (e.g., university websites, lectures)
- YouTube or other video platforms
- Books or e-books
- Other:

### When you hear the words “CRISPR Crops” please write down the first three words that come to mind. If you are unable to think of three, please provide as many as you can.

Please write your answer here:

### Do you think that GMOs and CRISPR crops are subject to the same regulations in Canada?

- Yes
- No
- Unsure

### Where available, how likely are you to purchase food edited by CRISPR/Cas9?

- Extremely unlikely
- Unlikely
- Neutral - neither unlikely or likely
- Likely
- Extremely likely

### Would you purchase CRISPR/Cas9 edited foods from the following categories? Please select all that apply:

- Seafood
- Fruits and Vegetables
- Dairy
- Poultry
- Beef
- Pork
- None of the above / I would not purchase

### Please rank your opinions on the following statements:

Please choose the appropriate response for each item:

I would consume crops or food genetically edited by CRISPR if they were cheaper than conventional products.

- Strongly Disagree
- Disagree
- Neither agree nor disagree
- Agree
- Strongly Agree

I would consume crops or food genetically edited by CRISPR if their nutritional quality was better than conventional products.

- Strongly Disagree
- Disagree
- Neither agree nor disagree
- Agree
- Strongly Agree

I would consume crops or food genetically edited by CRISPR if they were the same price as conventional products.

- Strongly Disagree
- Disagree
- Neither agree nor disagree
- Agree
- Strongly Agree

**Appendix B**

**Interview Guide**

1. Could you please describe your educational background, your path into this area of research, and your current involvement in the field?

2. What key themes or patterns have you noticed in the reporting on GMOs, and how have these themes influenced public perception and discourse?

3. How do you believe media coverage and reports of GMOs have or will influence public attitudes toward CRISPR crops?

a. What challenges do you foresee arising as these foods become available?

4.What has been your experience regarding public engagement and interaction with CRISPR crop technology?

a. How do you perceive the public’s receptiveness to this technology compared to GMOs?

5. Looking ahead, what trends do you anticipate in the media coverage of bio/genetic technology that could impact public understanding and acceptance of CRISPR crops in the future?

6. In what ways do you believe media and research outlets can better engage the public in discussions about GMOs, CRISPR crops, and similar related technologies?

**Appendix C**

Genetically Modified Organisms (GMOs) are plants or animals whose DNA has been changed using various techniques to improve certain traits. These methods often involve introducing genes from different species into the organism to enhance qualities like pest resistance or nutritional value. This allows scientists to create crops that can grow better and adapt to environmental challenges.

**Appendix D**

CRISPR/Cas9 is a gene-editing tool that allows scientists to make precise changes to DNA. It targets specific parts of the genetic code and cuts or modifies the DNA at those spots. This technology can improve crops by enhancing traits like nutritional value or pest resistance, offering a new way to address challenges in food production.
